# Prävention und Früherkennung in der Onkologie

**DOI:** 10.1007/s00108-026-02073-w

**Published:** 2026-03-05

**Authors:** Minna Voigtlaender, Judith Gebauer, Friederike Stoelzel, Barbara Kempf, Marianne Sinn, Nicole Skoetz

**Affiliations:** 1 Department of Oncology, Hematology and BMT with Section of Pneumology, University Medical Center Hamburg-Eppendorf, Hamburg, Deutschland; 2 German Society of Hematology and Medical Oncology (DGHO), Bauhofstraße 12, 10117 Berlin, Deutschland; 3 Department of Oncology and University Cancer Center Leipzig, University Hospital Leipzig, Leipzig, Deutschland; 4 University Cancer Center Schleswig-Holstein, University Hospital Schleswig-Holstein, Kiel, Deutschland; 5 Berliner Krebsgesellschaft e. V., Berlin, Deutschland; 6 Institut für Öffentliches Gesundheitswesen, Medizinische Fakultät und Uniklinik Köln, Universität zu Köln, Köln, Deutschland

**Keywords:** Krebsprävention, Primär‑, Sekundär und Tertiärprävention, Krebsscreening, Krebsüberleben, Deutsche Gesellschaft für Hämatologie und medizinische Onkologie (DGHO), Prevention in oncology, Primary, secondary and tertiary prevention, Cancer screening, Cancer survivorship, German Society of Hematology and Medical Oncology (DGHO)

## Abstract

In der Onkologie kommt der Prävention eine Schlüsselrolle zur Senkung von Krankheitsentstehung, Krankheitslast und Mortalität zu. Primär‑, Sekundär- und Tertiärprävention sind als komplementäre Ebenen zu verstehen, deren Potenzial bislang unzureichend ausgeschöpft ist. Die Primärprävention adressiert modifizierbare Risikofaktoren wie Tabak- und Alkoholkonsum, Adipositas, Bewegungsmangel, UV-Exposition und onkogene Infektionen. Schätzungen zufolge könnten in Europa rund 40 % der Krebsneuerkrankungen durch konsequente primärpräventive Maßnahmen vermieden werden. Die Sekundärprävention umfasst qualitätsgesicherte Krebsfrüherkennungsprogramme, deren Wirksamkeit belegt ist, deren Inanspruchnahme jedoch weiterhin begrenzt bleibt. Aktuelle Entwicklungen betreffen u. a. die Einführung einer organisierten Lungenkrebsfrüherkennung für definierte Hochrisikopopulationen. Mit der zunehmenden Zahl von Krebsüberlebenden gewinnt die Tertiärprävention weiter an Relevanz; strukturierte, multimodale und multiprofessionelle Nachsorge, Rehabilitation und Survivorship-Programme sind essenziell zur Reduktion von Rezidiven sowie langfristigen somatischen und psychosozialen Folgen. Eine wirksame Krebsprävention erfordert die systematische Verankerung evidenzbasierter Maßnahmen in Versorgung, Forschung und Gesundheitspolitik. Medizinisch-wissenschaftliche Fachgesellschaften leisten einen aktiven Beitrag zur strategischen Weiterentwicklung.

## Abstract (en)

Prevention plays a key role in reducing the development and burden of disease and mortality in oncology. Primary, secondary and tertiary prevention should be understood as complementary levels where the preventive potential has not yet been sufficiently exploited. Primary prevention addresses modifiable risk factors, such as tobacco and alcohol consumption, obesity, lack of exercise, UV exposure and oncogenic infections. According to estimates, around 40% of new cancer cases in Europe could be prevented through consistent primary prevention measures. Secondary prevention comprises quality-assured, established cancer screening programs where the effectiveness has been proven but the utilization remains limited. Current developments include the introduction of organized lung cancer screening for defined high-risk populations. With the increasing number of cancer survivors, tertiary prevention is becoming increasingly more relevant; structured, multimodal and multiprofessional aftercare, rehabilitation and survivorship programs are essential for reducing recurrences and long-term somatic and psychosocial consequences. An effective cancer prevention requires the systematic integration of evidence-based measures into care, research and health policy. Medical and scientific societies actively contribute to strategic development in this area.

## Einführung und Potenzial

Prävention ist in der Onkologie auf allen drei Präventionsebenen zentral, um Krankheitsentstehung, Krankheitslast, Mortalität und gesundheitsökonomische Folgekosten nachhaltig zu reduzieren. Primärprävention zielt auf die Verhinderung der Krebsentstehung, Sekundärprävention auf die frühzeitige Erkennung und Tertiärprävention auf die Vermeidung bzw. frühe Detektion von Rezidiven, aber auch Zweittumoren sowie die Reduktion somatischer und psychosozialer Langzeitfolgen.

Ein besonders großes Potenzial liegt in der Primärprävention: Nur etwa 5 % aller Krebserkrankungen sind durch singuläre hereditäre Keimbahnveränderungen bedingt, während die Mehrheit auf im Lebensverlauf erworbene genetische Veränderungen unter dem Einfluss exogener und endogener Faktoren beruht. Schätzungen zufolge sind rund 40 % der Krebsneuerkrankungen durch konsequente primärpräventive Maßnahmen vermeidbar [1].

Da viele Tumorerkrankungen bei früher Diagnose effektiver behandelt oder geheilt werden können, ist die Sekundärprävention von hoher Bedeutung. Qualitätsgesicherte Früherkennungsprogramme zielen auf die Identifikation von Krebserkrankungen in Stadien, in denen Therapieerfolg und langfristige Prognose deutlich verbessert sind.

Angesichts der demografischen Entwicklung und steigender Überlebensraten von Patientinnen und Patienten (Pat.) mit Krebs gewinnt die Tertiärprävention zunehmend an Bedeutung. Sie trägt zum langfristigen Erhalt von Lebensqualität und Leistungsfähigkeit von Krebsüberlebenden bei und ist integraler Bestandteil moderner onkologischer Versorgung.

Insgesamt sind die Präventionsebenen komplementär entlang des Krankheitsverlaufs zu verstehen; ihr Potenzial ist bislang jedoch nicht ausgeschöpft und erfordert eine systematische Verankerung in Versorgung, Forschung und Gesundheitspolitik.

## Evidenz und bisherige Anstrengungen

### Primärprävention

Die Primärprävention adressiert modifizierbare Krebsursachen auf individueller und bevölkerungsbezogener Ebene (Tab. 1).

**Tab. 1:** Evidenzbasierte Maßnahmen der primären Krebsprävention

Vermeidung des Tabakkonsums
Reduktion eines gesundheitsschädlichen Alkoholkonsums
Förderung einer ausgewogenen Ernährung
Förderung regelmäßiger körperlicher Aktivität
Vermeidung von Übergewicht und Adipositas
Schutz vor UV- und ionisierender Strahlung (einschließlich Radon)
Reduktion beruflicher Exposition gegenüber Karzinogenen
Prävention und Behandlung onkogener Infektionen (u. a. HPV, HBV)

Die Evidenz zu den in Tab. 1 aufgeführten Risikofaktoren ist robust. Tabakkonsum ist der wichtigste vermeidbare Einzelrisikofaktor und wird in Europa für rund 20 % aller Krebserkrankungen verantwortlich gemacht. Ein erhöhter BMI ist mit einem gesteigerten Risiko zahlreicher Krebserkrankungen, einschließlich hämatologischer Neoplasien, assoziiert [2]. Regelmäßige körperliche Aktivität erzielt protektive Effekte [3]. Infektionsassoziierte Malignome tragen weltweit wesentlich zur Krebsinzidenz bei; Impfprogramme gegen Humanes Papilloma Virus (HPV) und Hepatitis B (HBV) zählen zu den wirksamsten Interventionen zur Vermeidung HPV-assoziierter Tumoren (insbesondere der Anogenitalregion und des Oropharynx) sowie des HBV-assoziierten hepatozellulären Karzinoms.

Der weiterentwickelte European Code Against Cancer zielt auf Verhaltens- und Verhältnisprävention

Einen Rahmen für Primärprävention in Europa bildet der European Code Against Cancer, der auf Basis systematischer Evidenzbewertungen kontinuierlich weiterentwickelt wird [4]. Hervorzuheben ist die Berücksichtigung der Verhältnisprävention, da nachhaltige Effekte auf Bevölkerungsebene insbesondere dann zu erwarten sind, wenn gesundheitsförderliche Entscheidungen durch geeignete Rahmenbedingungen erleichtert werden. In Deutschland werden primärpräventive Maßnahmen hingegen eher an das Individuum im Sinne von Verhaltensprävention adressiert, indem jede Person die empfohlenen Präventionsansätze selbstverantwortlich berücksichtigen sollte.

Auf nationaler Ebene wurden in den letzten Jahren strukturelle Grundlagen zur stärkeren Verankerung der Krebsprävention gelegt. Mit dem Nationalen Krebspräventionszentrum wurde eine institutionelle Struktur für Forschung, Aus- und Weiterbildung, Öffentlichkeitsarbeit und strategische Weiterentwicklung geschaffen, im Zuge der Neuausrichtung des Nationalen Krebsplans wurde Primärprävention als Schwerpunkt verankert und im Rahmen der Nationalen Dekade gegen Krebs werden Forschungsaktivitäten zur Krebsprävention gezielt gefördert. Zudem wurden beim ersten Nationalen Krebspräventionsgipfels Handlungsempfehlungen zur Krebsprävention formuliert [5]. Auf Bundesebene fokussiert das Bundesinstitut für Öffentliche Gesundheit gesundheitliche Aufklärung.

### Sekundärprävention

In Deutschland wird die Sekundärprävention durch das gesetzliche Krebsfrüherkennungsprogramm adressiert (Tab. 2). Organisierte und opportunistische Angebote unterscheiden sich insbesondere durch systematische Einladungen und strukturierte Informationen, in denen Nutzen und Schaden neutral und mit absoluten Risiken präsentiert werden.

**Tab. 2:** Krebsfrüherkennungsuntersuchungen im Leistungsumfang der gesetzlichen Krankenversicherung (Lungenkrebsfrüherkennung folgt)

Organ	Alter (Jahre)	Frau	Mann	Häufigkeit	Struktur
Gebärmutterhals	20–34	Pap-Abstrich		Jährlich	Organisiert*
	Ab 35	Zusätzlich: HPV-Test		Alle 3 Jahre	Organisiert*
Brust	Ab 20	Tastuntersuchung		Jährlich	Opportunistisch***
	50–75	Mammographie		Alle 2 Jahre	Organisiert**
Darm	Ab 50	Zwei Koloskopien im Abstand von 10 Jahren			Organisiert*
		Stuhltest auf okkultes Blut alternativ zur Koloskopie		Alle 2 Jahre	Organisiert*
Haut	Ab 35	Ganzkörperinspektion der Haut		Alle 2 Jahre	Opportunistisch***
Prostata	Ab 45		Tastuntersuchung	Jährlich	Opportunistisch***

*Alle 5 Jahre Anschreiben durch Krankenkasse

**Schriftliche Einladung in eine Screeningeinheit, einschl. Entscheidungshilfe

***Anlassbezogen, initiiert von Pat. und Arzt/Ärztin

Bis 2019 wurde das Darmkrebsfrüherkennungsprogramm opportunistisch angeboten. Versichertendaten (2010–2022) zeigen eine geringe Nutzung stuhlbasierter Tests bei 50- bis 54-Jährigen (22,9 % der Männer, 55,5 % der Frauen) [6]. Populationsbasierte Effekte des organisierten Programms sind bislang nicht robust untersucht; Daten aus den Niederlanden zeigen mit einladungsbasierten Programmen, einschließlich Versand von Testkits und Erinnerungssystemen, Teilnahmeraten bis 70 %.

Das Mammographiescreening wurde 2009 als organisiertes Programm eingeführt und ist bei Teilnahme mit einer 20- bis 30 %igen relativen Reduktion der Brustkrebsmortalität assoziiert. Absolut betragen die Effekte auf die Gesamtmortalität 0,1–0,3 %. Auch hier ist eine informierte Entscheidung essenziell, um auf falsch-positive Ergebnisse und deren Folgen wie unnötigen Biopsien aufmerksam zu machen. Die Teilnahmequote war 2023 bei etwa 52 %. zu verzeichnen. In einer aktuellen bevölkerungsbasierten Gesundheitsstudie erwies sich die Inanspruchnahme anderer Präventionsmaßnahmen als stärkster Prädiktor für die Teilnahme [7].

Das Früherkennungsprogramm wird fortlaufend evidenzbasiert weiterentwickelt und ergänzt, zuletzt um die Einführung einer organisierten Lungenkrebsfrüherkennung mithilfe einer Niedrigdosis-CT für definierte Hochrisikogruppen. Die Grundlage bilden mehrere randomisierte Studien; exemplarisch zeigte die NELSON-Studie eine signifikante Reduktion der lungenkrebsspezifischen Mortalität (HR 0,76; 95 %-KI 0,61–0,94) sowie mehr Diagnosen in frühen Stadien der Karzinome [8]. Die Umsetzung als GKV-Leistung ist voraussichtlich ab April 2026 vorgesehen.

### Tertiärprävention

Ein zentrales Element ist die tumorspezifische strukturierte Surveillance zur Detektion von Rezidiven, Zweittumoren und Organtoxizität. Leitlinienbasierte Nachsorgestandards umfassen regelmäßige klinische Untersuchungen sowie labormedizinische und indikationsgerechte bildgebende Diagnostik. Die Evidenzlage ist uneinheitlich, und Empfehlungen können voneinander abweichen (z. B. Nachsorgeempfehlungen zum Kolonkarzinom der AWMF und ESMO [9]).

Ein strukturiertes Bewegungsprogramm kann das Überleben von Pat. nach Resektion eines Kolonkarzinoms verbessern

Die onkologische Rehabilitation bildet einen weiteren zentralen Versorgungsstrang. Sie adressiert körperliche, funktionelle und psychosoziale Defizite nach onkologischer Therapie und stellt häufig den ersten strukturierten Übergang in die Langzeitphase dar. Rehabilitationsmaßnahmen werden von rund einem Drittel der Pat. mit Krebs in Anspruch genommen, mit entitäts- und geschlechtsspezifischen Unterschieden.

Der Nutzen tertiärpräventiver Ansätze ist für ausgewählte Interventionen belegt. Beispielhaft zeigte eine aktuelle randomisierte Phase-III-Studie bei Pat. mit reseziertem Kolonkarzinom nach adjuvanter Chemotherapie, dass ein dreijähriges strukturiertes Trainingsprogramm (Ziel: körperliche Aktivität auf mindestens 10 MET-Stunden pro Woche, entsprechend etwa 150 min moderate Bewegung, zu steigern und über 3 Jahre zu halten) mit einem signifikant verlängerten krankheitsfreien (HR 0,72; 95 %-KI 0,55–0,94) und Gesamtüberleben (HR 0,63; 95 %-KI 0,43–0,94) assoziiert war [10].

### Cancer Survivorship

Der Begriff Cancer Survivorship bezeichnet Menschen mit und nach einer Krebserkrankung und umfasst ihre Perspektiven, Bedürfnisse und Gesundheit sowie die körperlichen, psychologischen, sozialen und ökonomischen Herausforderungen nach der Diagnose [11]. In Deutschland leben mindestens 4,5 Mio. Menschen mit oder nach Krebs; davon zwei Drittel als Langzeitüberlebende mit mindestens 5 Jahre zurückliegender Erstdiagnose.

Langzeit- und Spätfolgen umfassen u. a. Zweitmalignome, Kardiotoxizität, Polyneuropathien, Fertilitätsstörungen, Fatigue, depressive Symptome sowie berufliche und finanzielle Belastungen. Präventionsmaßnahmen fokussieren diese durch die Krebsbehandlung verursachten Erkrankungen und umfassen alle drei Ebenen (Tab. 3).

**Tab. 3:** Präventionsebenen und -maßnahmen im Cancer Survivorship-Kontext (modifiziert nach Ehrhardt et al. Nature Reviews Clinical Oncology 2023) bei Pat., die eine onkologische Behandlung erfahren

Präventionsebene	Maßnahme	Beispiele
Primär	Modifikation onkologischer Therapien zur Reduktion des Spätfolgenrisikos	Dexrazoxangabe bei Chemotherapie mit Anthrazyklinen
Sekundär	Spätfolgen-Früherkennung	Intensivierte Brustkrebsfrüherkennung nach thorakaler Strahlentherapie
Tertiär	Intervention im Frühstadium von Spätfolgen zur Prognoseverbesserung	Frühzeitiger Einsatz von Herzinsuffizienztherapie bei Kardiotoxizität

Multidimensionale Survivorship-Programme ergänzen Surveillance und Rehabilitation

Survivorship-Programme kombinieren leitliniengerechte Vorsorge- und Früherkennungsuntersuchungen mit evidenzbasierten Interventionen zu körperlicher Aktivität, gesunder Ernährung, psychosozialer Unterstützung und Fatigue-Management. Darüber hinaus beinhalten sie Angebote zur Förderung von Selbstmanagement, Gesundheitskompetenz und langfristiger Lebensstiladaption. Sie ergänzen Surveillance und Rehabilitation als multidimensionale Versorgungsmodelle.

Auf nationaler Ebene wird das Thema Langzeitüberleben nach Krebs sowohl im Rahmen des Nationalen Krebsplans als auch der Nationalen Dekade gegen Krebs systematisch adressiert.

## Defizite, Verbesserungsansätze und Zielsetzungen

### Primärprävention

Trotz hoher Evidenz und klar formulierter europäischer und nationaler Orientierungsrahmen ist das Potenzial der Primärprävention unzureichend ausgeschöpft. Zentrale Defizite liegen in der Umsetzung wirksamer, auf gut belegbaren Risikofaktoren beruhender Präventionsstrategien auf Bevölkerungsebene. Insbesondere verhältnispräventive Ansätze werden nicht systematisch, kohärent und mit ausreichender Reichweite implementiert. Voraussetzung hierfür ist die Verankerung von Prävention als Querschnittsaufgabe in allen Politikbereichen („Health-in-all-policies“).

Prävention ist als Querschnittsaufgabe in allen Politikbereichen zu verankern

Ein weiterer limitierender Faktor ist die unzureichende gesundheitsbezogene Risikokompetenz in Teilen der Bevölkerung. Evidenzbasierte Präventionsempfehlungen erreichen relevante Bevölkerungsgruppen nur eingeschränkt. Eine zentrale Aufgabe ist daher die frühzeitige, zielgruppenspezifische Förderung der Health Literacy (Gesundheitskompetenz).

Zudem war die Krebspräventionsforschung lange Zeit fragmentiert und strukturell unterrepräsentiert. In jüngerer Zeit wurden gezielt Forschungsverbünde etabliert, die die Kette von Risikoidentifikation über Intervention bis zur Evaluation adressieren. Erforderlich sind eine enge Verzahnung wissenschaftlicher Evidenz, gesundheitspolitischer Steuerung und Versorgungspraxis.

Schließlich erfordert die Weiterentwicklung der Primärprävention ein vertieftes Verständnis biologischer Mechanismen. Neben bekannten Karzinogenen rücken inflammatorische Prozesse in den Fokus, die die Akkumulation somatischer Mutationen begünstigen und die antitumorale Immunüberwachung beeinflussen können. Ziel sollte sein, hieraus differenzierte Risikoabschätzungen und perspektivisch individualisierbare Präventionsstrategien ableiten zu können.

### Sekundärprävention

Das Krebsfrüherkennungsprogramm ist strukturell etabliert und wird evidenzbasiert weiterentwickelt. Sein Potenzial wird durch eine unzureichende und sozial/strukturell heterogene Inanspruchnahme begrenzt. Zentrale Ziele sollten die nachhaltige Steigerung der Teilnahmequoten durch wirksame Einladungs- und Informationsstrategien, verbesserte Risikokommunikation sowie gezielte Forschung zu Teilnahme- und Zugangsdeterminanten sein.

Ziel zukünftiger Anstrengungen sollte eine risikoadaptierte Früherkennung sein

Darüber hinaus besteht ein wesentlicher Entwicklungsbedarf in der begrenzten Zielgruppenspezifität: Die bestehenden Programme differenzieren überwiegend ausschließlich nach Alter und Geschlecht. Ziel zukünftiger Anstrengungen sollte eine risikoadaptierte Früherkennung sein, die Hochrisikopopulationen präziser identifiziert und erreicht.

### Tertiärprävention

Auch in der tertiärpräventiven Versorgung bestehen weiterhin strukturelle Herausforderungen.

Insbesondere ist die Notwendigkeit des systematischen Transfers neuer Evidenz, auch zu nichtpharmakologischen Interventionen wie der Bewegungstherapie beim Kolonkarzinom, in qualitätsgesicherte Versorgungsprozesse zu nennen.

### Cancer Survivorship

Die Versorgung von Überlebenden nach Krebs ist per Definition multimodal und multiprofessionell und muss die unterschiedlichen Phasen (frühe Nachsorge, Langzeitnachsorge), Krankheitssituationen und individuellen Bedarf berücksichtigen. In der Versorgungspraxis erfolgt die Betreuung jedoch häufig entlang einzelner Versorgungssegmente, ohne dass Angebote systematisch abgestimmt sind. Nach Abschluss der meist 5‑jährigen Rezidivnachsorge fehlt oft eine strukturierte, auf Spätfolgen ausgerichtete Weiterbetreuung.

Spezialisierte Survivorship-Kliniken sind trotz positiver Entwicklungen nur an ausgewählten Standorten etabliert und versorgen bisher nur einen kleinen Anteil aller Krebsüberlebenden. Alternative bzw. ergänzende Versorgungskonzepte wurden nur vereinzelt und in der Regel als Pilotprojekte implementiert und tragen ebenfalls nicht relevant zur spezialisierten Versorgung dieser wachsenden Personengruppe teil.

Zentrale Entwicklungsziele sind die Etablierung koordinierter, sektorenübergreifender Survivorship-Versorgungsmodelle und deren Überführung in eine flächendeckende Regelversorgung. Damit verbunden ist der Aufbau standardisierter Datenerfassungs‑, Qualitäts- und Evaluationsstrukturen, um Versorgungspfade risikoadaptiert steuern und deren Wirksamkeit bewerten zu können.

## DGHO-Arbeitskreis Prävention

Vor dem Hintergrund der hohen Relevanz von Prävention über alle onkologischen Krankheitsphasen hinweg sieht sich die Deutsche Gesellschaft für Hämatologie und Medizinische Onkologie (DGHO) in besonderer fachlicher und gesellschaftlicher Verantwortung. Mit breiter Unterstützung der Mitglieder wurde zuletzt ein Arbeitskreis Prävention gegründet.

Ziele sind es, Prävention als integralen Bestandteil moderner Hämatologie und Onkologie zu stärken, den fachlichen Wissens- und Informationsaustausch zu fördern, interdisziplinäre Vernetzung und Synergien auszubauen, gemeinsame Forschungs- und Innovationsansätze zu entwickeln sowie die strategische Weiterentwicklung der Krebsprävention aktiv mitzugestalten.

## Schlussfolgerung

### Was ist wichtig?

- Primär‑, Sekundär- und Tertiärprävention sind zentrale Hebel zur Reduktion von Krebsinzidenz, -mortalität und Krankheitslast.
- Durch Primärprävention können rund 40 % der Krebsneuerkrankungen vermieden werden. Das Potenzial ist bislang unzureichend ausgeschöpft und erfordert koordinierte wissenschaftliche, gesellschaftliche und gesundheitspolitische Maßnahmen, einschließlich Verhältnisprävention.
- In Deutschland ist ein gesetzlich verankertes Krebsfrüherkennungsprogramm für Brust‑, Zervix‑, Darm‑, Prostata- und Hautkrebs etabliert, das evidenzbasiert weiterentwickelt wird. Die Teilnahme ist begrenzt, v. a. bei Menschen mit erhöhten Risiken für gesundheitliche Schäden. Zukünftige Forschung sollte risikoprofiladaptierte Strategien adressieren.
- Die Rolle tertiärpräventiver Maßnahmen nimmt mit der wachsenden Zahl der Krebsüberlebenden kontinuierlich zu. Survivorship-Programme fokussieren insbesondere auf Früherkennung neuer Erkrankungen (Spätfolgen) und benötigen koordinierte, multiprofessionelle und sektorenübergreifende Versorgungsmodelle.
- Nationale Initiativen und gezielte Forschungsförderung bilden einen wichtigen Rahmen für Weiterentwicklung, Vernetzung und Implementierung präventiver Strategien.
- Fachgesellschaften wie die Deutsche Gesellschaft für Hämatologie und Medizinische Onkologie (DGHO) übernehmen eine aktive Rolle bei der strategischen Weiterentwicklung der Krebsprävention.
